# Vitamin D and Caudal Primary Motor Cortex: A Magnetic Resonance Spectroscopy Study

**DOI:** 10.1371/journal.pone.0087314

**Published:** 2014-01-31

**Authors:** Cedric Annweiler, Olivier Beauchet, Robert Bartha, Vladimir Hachinski, Manuel Montero-Odasso, on behalf of the WALK Team (Working group Angers-London for Knowledge)

**Affiliations:** 1 Gait and Brain Lab, Lawson Health Research Institute, Parkwood Hospital, The University of Western Ontario, London, Ontario, Canada; 2 Division of Geriatric Medicine, University Hospital; Memory Clinic; UPRES EA 4638, University of Angers, Angers, France; 3 Robarts Research Institute, the University of Western Ontario, London, Ontario, Canada; 4 Department of Clinical Neurological Sciences, University Hospital, the University of Western Ontario, London, Ontario, Canada; University of Queensland, Australia

## Abstract

**Background:**

Vitamin D is involved in brain physiology and lower-extremity function. We investigated spectroscopy in a cohort of older adults to explore the hypothesis that lower vitamin D status was associated with impaired neuronal function in caudal primary motor cortex (cPMC) measured by proton magnetic resonance spectroscopic imaging.

**Methods:**

Twenty Caucasian community-dwellers (mean±standard deviation, 74.6±6.2 years; 35.0% female) from the ‘Gait and Brain Study’ were included in this analysis. Ratio of *N*-acetyl-aspartate to creatine (NAA/Cr), a marker of neuronal function, was calculated in cPMC. Participants were categorized according to mean NAA/Cr. Lower vitamin D status was defined as serum 25-hydroxyvitamin D (25OHD) concentration <75 nmol/L. Age, gender, number of comorbidities, vascular risk, cognition, gait performance, vitamin D supplements, undernourishment, cPMC thickness, white matter hyperintensities grade, serum parathyroid hormone concentration, and season of evaluation were used as potential confounders.

**Results:**

Compared to participants with high NAA/Cr (n = 11), those with low NAA/Cr (i.e., reduced neuronal function) had lower serum 25OHD concentration (P = 0.044) and more frequently lower vitamin D status (P = 0.038). Lower vitamin D status was cross-sectionally associated with a decrease in NAA/Cr after adjustment for clinical characteristics (β = −0.41, P = 0.047), neuroimaging measures (β = −0.47, P = 0.032) and serum measures (β = −0.45, P = 0.046).

**Conclusions:**

Lower vitamin D status was associated with reduced neuronal function in cPMC. These novel findings need to be replicated in larger and preferably longitudinal cohorts. They contribute to explain the pathophysiology of gait disorders in older adults with lower vitamin D status, and provide a scientific base for vitamin D replacement trials.

## Introduction

Beyond its long-known involvement in bone health, vitamin D has also emerged as a secosteroid hormone with non-skeletal effects [1], [2]. In particular, vitamin D is involved in neurophysiology [3],[4] and lower vitamin D status has been associated with whole-brain atrophy in older adults [5]. However, the specific brain regions that are altered with lower serum 25-hydroxyvitamin D (25OHD) concentrations remain unknown [6]. Based on the repeated findings that lower vitamin D status is associated with poor lower-extremity function in older adults, such as gait and balance disorders [7] with consequent falls and fractures [8], it appears likely that lower vitamin D status may negatively affect the brain structures involved in lower-limb motor control. Since the primary motor cortex (PMC) is the final integrator of motor control via a somatotopic organization [9,10), we hypothesized that lower vitamin D status in older adults could directly affect the subregion of the PMC responsible for lower-limb motricity (i.e., caudal PMC, cPMC). Our objective was to determine whether lower vitamin D status in older adults was associated with impaired neuronal function in cPMC measured by proton magnetic resonance spectroscopy (^1^H-MRS).

## Methods

### Participants

Baseline data from 20 consecutive participants recruited in the ‘Gait and Brain Study’ from September 2011 to March 2012 was used for this analysis. This cohort is being followed to prospectively evaluate the mobility declines in older adults with prodromal cognitive decline. The sampling and data collection procedures have been described elsewhere [11].

### ^1^H-MRS Acquisition

MR data were acquired on 3-Tesla Siemens Tim Trio MRI (Siemens, Erlangen, Germany), using 32-channel head coil. Each exam included the acquisition of sagittal 3D T_1_-weighted MP-RAGE anatomical images (repetition time/echo time = 2300/2.9 ms, inversion time = 900 ms, flip angle = 9°, averages = 1, FOV = 256×240×192 mm, matrix = 256×240×160) covering the entire brain.

Anatomical images guided the placement of a 20 mm isotropic voxel on leg and foot regions of right PMC. Both water suppressed (averages = 192) and unsuppressed (averages = 8) spectra were localized by point resolved spectroscopy (PRESS, repetition time/echo time = 2000/135 ms, voxel size = 8 cm^3^). Data processing included lineshape correction and removal of residual unsuppressed water signal [10], [12]. Resultant metabolite spectra were fitted in the time domain incorporating a template of prior knowledge of metabolite lineshapes including *N*-acetylaspartate (NAA) and creatine (Cr). NAA level is linked to the functional status of neuronal mitochondria and is considered a marker of neuronal function [13). Cr provides a measure of oxidative energy stores [14]. Therefore, NAA/Cr ratio provides a reproducible and sensitive measurement of neuronal integrity that also reduces quantification errors associated with differences in tissue partial volume between voxels. In our sample, participants were separated into two groups using a threshold NAA/Cr ratio of the mean value (i.e., NAA/Cr = 1.17).

### Serum Vitamin D Concentration

Venous blood was collected from resting participants at the time of brain assessments. Serum 25OHD concentration, an effective indicator of vitamin D status, was measured by radioimmunoassay (DiaSorin, IncstarCorp, Stillwater, MN). The intra- and interassay precisions were 5.2% and 11.3% respectively. Lower vitamin D status was defined as 25OHD concentrations <75 nmol/L (to convert to ng/mL, divide by 2.496) [2], [15]. All measurements were performed at the University of Western Ontario, London, ON.

### Confounders

Age, gender, number of comorbidities, vascular risk, global cognitive performance, high-level gait performance, use of vitamin D supplements, undernourishment, cPMC thickness, white matter hyperintensities (WMH) grade, serum parathyroid hormone (PTH) concentration, and season of evaluation were used as potential confounders.

Comorbidities were defined as diseases lasting at least 3 months or running a course with minimal changes. Vascular risk was assessed using the 7-point Vascular Factors Index [16]. Global cognition was assessed using the Montreal Cognitive Assessment (MoCA) [17]. High-level gait performance was estimated from stride time variability (STV), a valid marker of cortical control of gait [7], and measured with a computerized walkway (GAITRite®, CIR Systems, Havertown, PA) while steady-state walking and counting aloud backwards by 7 starting from 100 [11]. The use of vitamin D supplements was reported by direct inquiry, whatever the dosage schedule or route of administration, and regardless of the date of commencement. Undernourishment was defined as body mass index <21 kg/m^2^ [18]. The average cPMC thickness, calculated as the average distance between gray matter/white matter boundary and gray matter/cerebrospinal fluid boundary within the cPMC, was obtained from 3D T_1_-weighted MP-RAGE images using FreeSurfer (v5.1.0), a set of tools that automatically segments, labels and quantifies brain tissue volumes [19]. WMH grade was measured using a semiquantitative visual rating scale of 0–9 (worst) [20] applied to T_2_-weighted FLAIR images (acquisition matrix = 256×232, reconstructed to 512×464 matrix, FOV = 220 mm×200 mm, thickness = 4 mm, gap = 0.5 mm, 41 slices, TR = 8 s, TE = 120 ms, TI = 2400 ms, flip angle = 130 degrees, averages = 1). Serum concentration of intact PTH was measured by enzyme-linked immunosorbent assay (ALPCO Diagnostics, Salem, NH). Finally, the season of evaluation was recorded as follows: spring from March 21 to June 20, summer from June 21 to September 20, fall from September 21 to December 20, winter from December 21 to March 20.

### Statistical Analysis

Comparisons between participants separated into two groups based on NAA/Cr in cPMC (≤1.17 or >1.17) were performed using two-sided Mann-Whitney U-test or Chi-square test, as appropriate. Multivariate linear regressions were used to examine the association between lower vitamin D status (independent variable) and NAA/Cr in cPMC (dependent variable), while adjusting for clinical characteristics, neuroimaging measures, and serum measures. P-values<0.05 were considered significant. All statistics were performed using SPSS (v19.0, IBM Corporation, Chicago, IL).

### Ethics Statement

Participants in the study were included after having given their written informed consent for research. The study was conducted in accordance with the ethical standards set forth in the Helsinki Declaration (1983). The project was approved by the University of Western Ontario Research Ethics Board for Health Sciences Research Involving Human Subjects.

## Results

Among 20 participants (mean±standard deviation, 74.6±6.2 years; 13 males and 7 females), the mean 25OHD concentration was 110.8±45.4 nmol/L. Fifteen percent of the sample had lower vitamin D status (mean, 59.3±12.9 nmol/L). The mean NAA/Cr in cPMC was 1.17 among the whole group. Participants with NAA/Cr <1.17 (n = 9) had lower 25OHD concentration than those with NAA/Cr >1.17 (104.2±66.7 mmol/L versus 116.2±17.2 mmol/L, P = 0.044) and more frequently lower vitamin D status (P = 0.038) (Table 1). They also had higher (i.e., worse) STV (P = 0.030).

**Table 1:** Characteristics and comparison of participants (n = 20) separated into two groups based on NAA/Cr in cPMC.

		NAA/Cr in cPMC		
	Total sample(n = 20)	≤1.17(n = 9)	>1.17(n = 11)	P-Value*
**Clinical characteristics**				
Age, years	74.6±6.2	73.9±7.0	75.1±5.8	0.703
Female, n (%)	7 (35.0)	4 (44.4)	3 (27.3)	0.423
Number of comorbidities	5.7±2.6	5.9±2.9	5.6±2.5	0.969
Vascular risk index,/7	1.3±0.9	1.5±0.8	1.2±1.0	0.452
Montreal Cognitive Assessment score,/30	24.8±2.6	25.4±2.6	24.2±2.6	0.295
Stride time variability†, %	3.9±2.4	4.0±2.9	3.7±2.0	**0.030**
Use of vitamin D supplements, n (%)	11 (55.0)	6 (66.7)	5 (45.5)	0.343
Undernourishment‡, n (%)	2 (10)	2 (18.2)	0 (0.0)	0.178
**Neuroimaging measures**				
NAA/Cr in cPMC	1.17±0.19	1.03±0.13	1.29±0.14	**<0.001**
cPMC thickness, mm	4.54±0.43	4.50±0.44	4.58±0.44	0.870
White matter hyperintensities grade^||^,/9	2.5±1.6	2.9±1.7	2.1±1.5	0.198
**Serum measures**				
25-hydroxyvitamin D concentration, nmol/L	110.8±45.4	104.2±66.7	116.2±17.2	**0.044**
Lower vitamin D status§, n (%)	3 (15.0)	3 (33.0)	0 (0.0)	**0.038**
Parathyroid hormone concentration, pg/mL	27.5±19.2	34.1±25.1	22.0±11.2	0.110
**Season of evaluation** ¶				0.953
Spring, n (%)	4 (20)	2 (22.2)	2 (18.2)	
Summer, n (%)	5 (25)	2 (22.2)	3 (27.3)	
Fall, n (%)	8 (40)	4 (44.4)	4 (36.4)	
Winter, n (%)	3 (15)	1 (11.1)	2 (18.2)	

Data presented as mean±standard deviation where applicable. Cr: creatine; cPMC: caudal primary motor cortex; NAA: *N-*acetylaspartate;

* Comparisons between participants with NAA/Cr≤1.17 and participants with NAA/Cr>1.17 based on Mann-Whitney U-test or Chi-square, as appropriate;

† Measured while steady-state walking and counting backwards by seven;

‡ body mass index <21 kg/m^2^;

||Manolio scale;

§ Serum 25-hydroxyvitamin D <75 nmol/L;

¶ Spring from 21 March to 20 June; Summer from 21 June to 20 September; Fall from 21 September to 20 December; and Winter from 21 December to 20 March; P significant (i.e. <0.05) indicated in bold.

Multivariate linear regression models showed that lower vitamin D status was associated with a decrease in NAA/Cr in cPMC (β = −0.45, P = 0.046) (Table 2) and with MoCA score (β = −0.08, P = 0.047).

**Table 2:** Multivariate linear regression examining the cross-sectional association between NAA/Cr in cPMC and lower vitamin D status*, adjusted for potential confounders† ^.^

	NAA/Cr in the cPMC								
	Model 1			Model 2			Model 3		
	β	95%CI	P-Value	β	95%CI	P-Value	β	95%CI	P-Value
Lower vitamin D status*	−**0.41**	−**0.82;** −**0.10**	**0.047**	−**0.47**	−**0.88;** −**0.06**	**0.032**	−**0.45**	−**0.90;** −**0.01**	**0.046**
**Clinical characteristics**									
Age	−0.01	−0.02;0.02	0.671	0.01	−0.01;0.03	0.285	0.01	−0.02;0.04	0.534
Female gender	0.03	−0.23;0.30	0.787	−0.04	−0.32;0.23	0.717	−0.03	−0.33;0.27	0.779
Number of comorbidities	−0.01	−0.06;0.05	0.945	−0.01	−0.08;0.06	0.966	0.02	−0.09;0.13	0.636
Vascular risk index	0.11	−0.04;0.27	0.121	0.12	−0.11;0.36	0.240	0.10	−0.17;0.36	0.359
Montreal CognitiveAssessment score	−**0.05**	−**0.10;** −**0.01**	**0.048**	−**0.06**	−**0.11;** −**0.01**	**0.032**	−**0.08**	−**0.16;** −**0.01**	**0.047**
STV‡	−0.01	−0.04;0.03	0.723	0.01	−0.03;0.04	0.734	0.01	−0.03;0.06	0.436
Use of vitamin D supplements	−0.16	−0.41;0.09	0.186	−0.15	−0.45;0.14	0.242	0.02	−0.55;0.59	0.926
Undernourishment^||^	0.05	−0.31;0.40	0.770	−0.01	−0.36;0.33	0.920	0.10	−0.39;0.59	0.592
**Neuroimaging measures**									
cPMC thickness	−	−	−	0.32	−0.05;0.69	0.079	0.42	−0.07;0.91	0.076
White matter hyperintensitiesgrade§	−	−	−	−0.01	−0.10;0.10	0.949	0.03	−0.11;0.17	0.583
**Serum measures**									
Parathyroid hormone concentration	−	−	−	−	−	−	−0.10	−0.04;0.02	0.371

Model 1: adjusted for clinical characteristics; Model 2: model 1+adjustment for neuroimaging measures; Model 3: model 2+ adjustment for serum measures; β: coefficient of regression corresponding to a change of NAA/Cr in cPMC; CI: confidence interval; Cr: creatine; cPMC: caudal primary motor cortex; NAA: *N-*acetylaspartate;

* serum 25-hydroxyvitamin D <75 nmol/L;

† all models included the influence of seasons with no significant effect (P = 0.942 for Model 1; P = 0.394 for Model 2; P = 0.247 for Model 3);

‡ measured while steady-state walking and counting backwards by seven;

||body mass index <21 kg/m^2^;

§ Manolio scale; β significant indicated in bold.

## Discussion

To our knowledge, this study provides the first evidence of an association between lower vitamin D status and reduced cPMC function illustrated by decreased NAA/Cr.

The PMC, located in the precentral gyrus of the cerebral cortex (Brodmann area 4), is the final integrator of all brain processes involved in gait control before the descending corticospinal tract [9], [10]. Thus, finding that lower vitamin D status was associated with decreased NAA/Cr in cPMC has potential clinical implications. Locomotion disorders accompanying lower vitamin D status have been explained mainly by an adverse impact on striated muscles [21]; however, a recent meta-analysis highlighted that such muscular effects of vitamin D remain uncertain [8], paving the way for new working hypotheses. We propose that lower vitamin D status could influence lower-extremity function by affecting brain structures responsible for higher-level motor control. In fact, this hypothesis is consistent with previous studies reporting that transgenic mice lacking functional vitamin D receptors (VDRs) in the brain exhibited uncoordinated lower-limb movements [22].

The mechanism linking vitamin D with cPMC function is not firmly established. Vitamin D is a lipophilic molecule that crosses the blood-brain barrier and exerts action through VDRs present in cortical neurons [2]–[4]. Vitamin D regulates the gene expression of several neurotrophins and protects neural networks by controlling mitosis rate and neuronal growth [4], [5]. Animal experiments have also shown that vitamin D regulates intra-neuronal calcium homeostasis, and oxidative and inflammatory changes in the brain [3], [4], promoting neuron viability and function. However, causality cannot be determined from our cross-sectional study. It may be argued that, contrary to our hypothesis, reduced neuronal function in cPMC could result in lower vitamin D status due to lower-extremity dysfunction with poor mobility and low sun exposure. However, this hypothesis should be mitigated by the high functionality of our sample, all scoring 6 out of 6 on Katz’s Activities of Daily Living score.

Moreover, although we were able to control for important characteristics that could modify the association, residual potential confounders such as serum concentrations of calcium and phosphorus might still be present.

We also found a significant inverse association, although of low magnitude, between MoCA score and NAA/Cr in the cPMC. This result is consistent with prior transcranial magnetic stimulation/electroencephalogram studies reporting a link between cognition and PMC, specifically that cognitively preparing a movement results in an anticipatory reduction of PMC excitability [23].

## Conclusions

In conclusion, we report an association between lower vitamin D status and decreased NAA/Cr –indicative of reduced neuronal function– in the cPMC. Even if these novel findings need to be replicated in larger and preferably longitudinal cohorts, they contribute to explain the pathophysiology of higher-level gait disorders in older adults with lower vitamin D status.

## References

[1] Holick MF (2007) Vitamin D deficiency. N Engl J Med 357: 266–281. 17634462 10.1056/NEJMra070553

[2] Annweiler C, Souberbielle JC, Schott AM, de Decker L, Berrut G, Beauchet O (2011) Vitamin D in the elderly: 5 points to remember. Geriatr Psychol Neuropsychiatr Vieil 9: 259–267. 21896429 10.1684/pnv.2011.0288

[3] Kalueff AV, Tuohimaa P (2007) Neurosteroid hormone vitamin D and its utility in clinical nutrition. Curr Opin Clin Nutr Metab Care 10: 12–19. 17143049 10.1097/MCO.0b013e328010ca18

[4] Annweiler C, Schott AM, Berrut G, Chauviré V, Le Gall D, et al (2010) Vitamin D and Ageing: Neurological issues. Neuropsychobiology 62: 139–150. 20628264 10.1159/000318570

[5] Annweiler C, Montero-Odasso M, Hachinski V, Seshadri S, Bartha R, Beauchet O (2013) Vitamin D concentration and lateral cerebral ventricle volume in older adults. Mol Nutr Food Res 57: 267–276. 23281306 10.1002/mnfr.201200418

[6] Annweiler C, Montero-Odasso M, Muir SW, Beauchet O (2012) Vitamin D and brain imaging in the elderly: Should we expect some lesions specifically related to hypovitaminosis D? Open Neuroimag J 6: 16–18. 22423308 10.2174/1874440001206010016 PMC3296113

[7] Beauchet O, Annweiler C, Verghese J, Fantino B, Herrmann FR, Allali G (2011) Biology of gait control: vitamin D involvement. Neurology 76: 1617–1622. 21471466 10.1212/WNL.0b013e318219fb08 PMC3100089

[8] Muir SW, Montero-Odasso M (2011) Effect of vitamin D supplementation on muscle strength, gait and balance in older adults: a systematic review and meta-analysis. J Am Geriatr Soc 59: 2291–2300. 22188076 10.1111/j.1532-5415.2011.03733.x

[9] Meier JD, Aflalo TN, Kastner S, Graziano MSA (2008) Complex organization of human primary motor cortex: A high-resolution fMRI study. J Neurophysiol 100: 1800–1812. 18684903 10.1152/jn.90531.2008 PMC2576195

[10] Annweiler C, Beauchet O, Bartha R, Wells JL, Borrie MJ, et al (2013) Motor cortex and gait in mild cognitive impairment: a magnetic resonance spectroscopy and volumetric imaging study. Brain 136: 859–871. 23436505 10.1093/brain/aws373

[11] Montero-Odasso M, Casas A, Hansen KT, Bilski P, Gutmanis I, et al (2009) Quantitative gait analysis under dual-task in older people with mild cognitive impairment: a reliability study. J Neuroeng Rehabil 6: 35. 19772593 10.1186/1743-0003-6-35 PMC2754991

[12] Bartha R, Drost DJ, Williamson PC (1999) Factors affecting the quantification of short echo in vivo 1H MR spectra: prior knowledge, peak elimination and filtering. NMR Biomed 12: 205–216. 10421912 10.1002/(sici)1099-1492(199906)12:4<205::aid-nbm558>3.0.co;2-1

[13] Moffett JR, Ross B, Arun P, Madhavarao CN, Namboodiri AM (2007) N-Acetylaspartate in the CNS: from neurodiagnostics to neurobiology. Prog Neurobiol 81: 89–131. 17275978 10.1016/j.pneurobio.2006.12.003 PMC1919520

[14] Wozniak JR, Lim KO (2006) Advances in white matter imaging: a review of in vivo magnetic resonance methodologies and their applicability to the study of development and aging. Neurosci Biobehav Rev 30: 762–774. 16890990 10.1016/j.neubiorev.2006.06.003 PMC2895765

[15] Holick MF, Binkley NC, Bischoff-Ferrari HA, Gordon CM, Hanley DA, Heaney RP, et al (2011) Evaluation, treatment, and prevention of vitamin D deficiency: an Endocrine Society clinical practice guideline. J Clin Endocrinol Metab 96: 1911–1930. 21646368 10.1210/jc.2011-0385

[16] Villeneuve S, Belleville S, Massoud F, Bocti C, Gauthier S (2009) Impact of Vascular Risk Factors and Diseases on Cognition in Persons with Mild Cognitive Impairment. Dement Geriatr Cogn Disord 27: 375–381. 19321941 10.1159/000209965

[17] Nasreddine ZS, Phillips NA, Bédirian V, Charbonneau S, Whitehead V, et al (2005) The Montreal Cognitive Assessment, MoCA: A brief screening tool for mild cognitive impairment. J Am Geriatr Soc 53: 695–699. 15817019 10.1111/j.1532-5415.2005.53221.x

[18] Management strategy of protein-energy malnutrition in the elderly. French National Authority for Health (online). Available at: www.hassante.fr/portail/jcms/c_546549/strategie-de-prise-en-charge-en-cas-de-denutritionproteino-energetique-chez-la-personne-agee. Accessed November 1st, 2013.

[19] Fischl B, Salat DH, Busa E, Albert M, Dieterich M, Haselgrove C, et al (2002) Whole brain segmentation: automated labeling of neuroanatomical structures in the human brain. Neuron 33: 341–355. 11832223 10.1016/s0896-6273(02)00569-x

[20] Manolio TA, Kronmal RA, Burke GL, Poirier V, O’Leary DH, et al (1994) Magnetic resonance abnormalities and cardiovascular disease in older adults: the Cardiovascular Health Study. Stroke 25: 318–327. 8303738 10.1161/01.str.25.2.318

[21] Annweiler C, Montero-Odasso M, Schott AM, Berrut G, Fantino B, Beauchet O (2010) Fall prevention and vitamin D in the elderly: an overview of the key role of the non-bone effects. J Neuroeng Rehabil 7: 50. 20937091 10.1186/1743-0003-7-50 PMC2959005

[22] Burne TH, Johnston AN, McGrath J, Mackay-Sim A (2006) Swimming behaviour and post-swimming activity in Vitamin D receptor knockout mice. Brain Res Bull 69: 74–78. 16464687 10.1016/j.brainresbull.2005.10.014

[23] Bonnard M, Spieser L, Meziane HB, de Graaf J, Pailhous J (2008) How cognition can influence the excitability of the primary motor cortex? A TMS-EEG study. Brain Stimulation 1: 274.

